# Molecular Characterization Informs Prognosis in Patients With Localized Ewing Sarcoma: A Report From the Children's Oncology Group

**DOI:** 10.1200/JCO-25-00157

**Published:** 2025-11-03

**Authors:** Riaz Gillani, David S. Shulman, Natalie J. DelRocco, Kelly Klega, Ruxu Han, Mark D. Krailo, Jonathan C. Slack, Mohammad Tanhaemami, Abigail Ward, Victoria Bainer, Cora Ricker, Josee Sparks, Kelly M. Bailey, Damon R. Reed, Steven G. DuBois, Patrick Leavey, Leo Mascarenhas, Patrick J. Grohar, Alanna J. Church, Brian D. Crompton, Katherine A. Janeway

**Affiliations:** ^1^ Department of Pediatric Oncology, Dana-Farber Cancer Institute, Boston, MA; ^2^ Harvard Medical School, Boston, MA; ^3^ Boston Children's Hospital, Boston, MA; ^4^ Cancer Program, Broad Institute of Harvard and MIT, Cambridge, MA; ^5^ Children's Oncology Group, Monrovia, CA; ^6^ Department of Population and Public Health Sciences, Keck School of Medicine, University of Southern California, Los Angeles, CA; ^7^ Robert J. Tomsich Institute of Pathology and Laboratory Medicine, Cleveland Clinic, Cleveland, OH; ^8^ Biopathology Center, Abigail Wexner Research Institute at Nationwide Children's Hospital, Columbus, OH; ^9^ University of Pittsburgh School of Medicine, Pittsburgh, PA; ^10^ Department of Pediatrics, Memorial Sloan Kettering Cancer Center, New York, NY; ^11^ Department of Medicine, Memorial Sloan Kettering Cancer Center, New York, NY; ^12^ Department of Pediatrics, University of Texas Southwestern Medical Center, Dallas, TX; ^13^ Children's Medical Center, Dallas, TX; ^14^ Cedars-Sinai Medical Center, Los Angeles, CA; ^15^ Department of Pediatrics, Division of Pediatric Hematology Oncology, University of Michigan Medical School, CS Mott Children's Hospital, Ann Arbor, MI

## Abstract

**PURPOSE:**

Identifying discrete subgroups associated with treatment response and resistance in localized Ewing sarcoma (EWS) remains a challenge. The primary objective of the Children's Oncology Group (COG) biology study AEWS18B1-Q was to molecularly characterize patients with localized EWS on prospective modern-day trials.

**PATIENTS AND METHODS:**

We analyzed clinical and molecular features from patients with localized EWS enrolled on frontline COG trials. All patients had available formalin-fixed paraffin-embedded (FFPE) tissue, frozen tissue, or whole-genome–amplified material. Sequencing was performed for identification of canonical fusions, recurrent copy number alterations (CNAs), and alterations in *TP53* and *STAG2*. Available tissue was analyzed for loss of STAG2 protein expression. Molecular features were evaluated for their association with cumulative incidence of relapse in univariate and multivariable analyses.

**RESULTS:**

Three hundred fifty-one patients had sufficient tissue, which in most cases was extracted from two FFPE slides. EWS canonical fusions were identified in 282 patients (80.3%). Pathogenic mutations in *TP53* and *STAG2* were identified in 5.1% and 7.6% of patients, respectively. A total of 63.1% of patients were found to have recurrent CNAs. In univariate analysis, there was an increased cumulative incidence of relapse in patients with *TP53* mutation (5-year cumulative incidence of relapse 43%, 95% CI [17% to 67%] *v* 22%, 95% CI [17% to 27%]; Gray's test *P* = .039), *STAG2* mutation (53%, 95% CI [29% to 73%] *v* 21%, 95% CI [16% to 26%]; *P* < .001), and recurrent CNAs (30%, 95% CI [22% to 37%] *v* 16%, 95% CI [9% to 24%]; *P* = .005). In a multivariable analysis, *STAG2* mutation was the only molecular biomarker that remained prognostic.

**CONCLUSION:**

This is a prospective validation of the molecular prognostic features of patients with localized EWS receiving standard-of-care therapy on therapeutic clinical trials. Building on previous work, patients with *STAG2* mutations were at high risk of relapse.

## INTRODUCTION

Ewing sarcoma (EWS) is an aggressive bone and soft tissue sarcoma defined by the presence of FET-ETS family fusions.^1^ The estimated 5-year event-free survival (EFS) is 78% for the approximately 70% of patients who present with localized disease enrolled on the most recent Children's Oncology Group (COG) phase 3 trial with a treatment backbone of interval-compressed (every 2 weeks) chemotherapy.^2^ Survivors experience late effects including cardiac dysfunction, second malignant neoplasms, infertility, and physical disability.^3-5^ There is therefore an urgent need to define molecularly characterized risk groups to inform approaches to risk-stratified therapy, as has been done in the context of other solid tumors and hematologic malignancies,^6,7^ with the goal of improving cure rates for high-risk patients and reducing morbidity among patients with less aggressive disease.

**CONTEXT**

**Key Objective**
To determine whether molecular features can identify clinically relevant disease subgroups applicable to frontline clinical trial design among patients with localized Ewing sarcoma (EWS).
**Knowledge Generated**
Among 351 patients with localized EWS treated on prospective trials, canonical fusions were identified in 80% of patients. In a multivariable analysis of patients with genomically defined EWS, the presence of *STAG2* mutations identified a high-risk population.
**Relevance *(S. Bhatia)***
This study provides evidence for the incorporation of an integrated molecular biomarker strategy to risk-stratify treatment strategies in patients with localized EWS.*
*Relevance section written by *JCO* Associate Editor Smita Bhatia, MD, MPH, FASCO.

Clinical features alone have proven to be inadequate to risk stratify patients with localized EWS.^8,9^ A growing body of evidence dating back to initial genomic landscape studies has contributed to our understanding of key molecular features of EWS.^10-12^ Previous studies have suggested that inactivation of *STAG2* and/or *TP53* may be associated with poor prognosis.^13,14^ Similarly, multiple recurrent copy number alterations (CNAs) have preliminary evidence for association with poor outcomes.^12,15,16^ These molecular biomarkers hold the potential to define prognostic subgroups in EWS but require clinical validation in independent patient cohorts with prospective outcome data collection.^17^

Thus, given the emerging evidence for multiple prognostic molecular biomarkers in EWS, we pursued multimodal molecular analysis of a large cohort of patients with localized EWS treated with contemporary therapy on COG clinical trials. We sought to (1) search for disease-defining translocations, (2) describe the prevalence of relevant molecular biomarkers in a large cohort of patients with molecularly defined localized EWS, and (3) identify molecularly informed disease subgroups by testing for associations between molecular features (eg, *STAG2* and *TP53* alterations) and outcomes.

## PATIENTS AND METHODS

Patients were required to have a pathologic diagnosis of EWS and be enrolled on and eligible for frontline clinical trials AEWS0031, AEWS1031, or INT-0154. Targeted panel sequencing, ultralow pass whole-genome sequencing , and immunohistochemistry (IHC) were used for molecular characterization of the study cohort, and these data were combined with clinical and survival data. Additional details pertaining to methods are given in the Data Supplement (Appendix A and Appendix Tables A1 and A2, online only).

## RESULTS

### Patients and Samples

**FIG 1.**
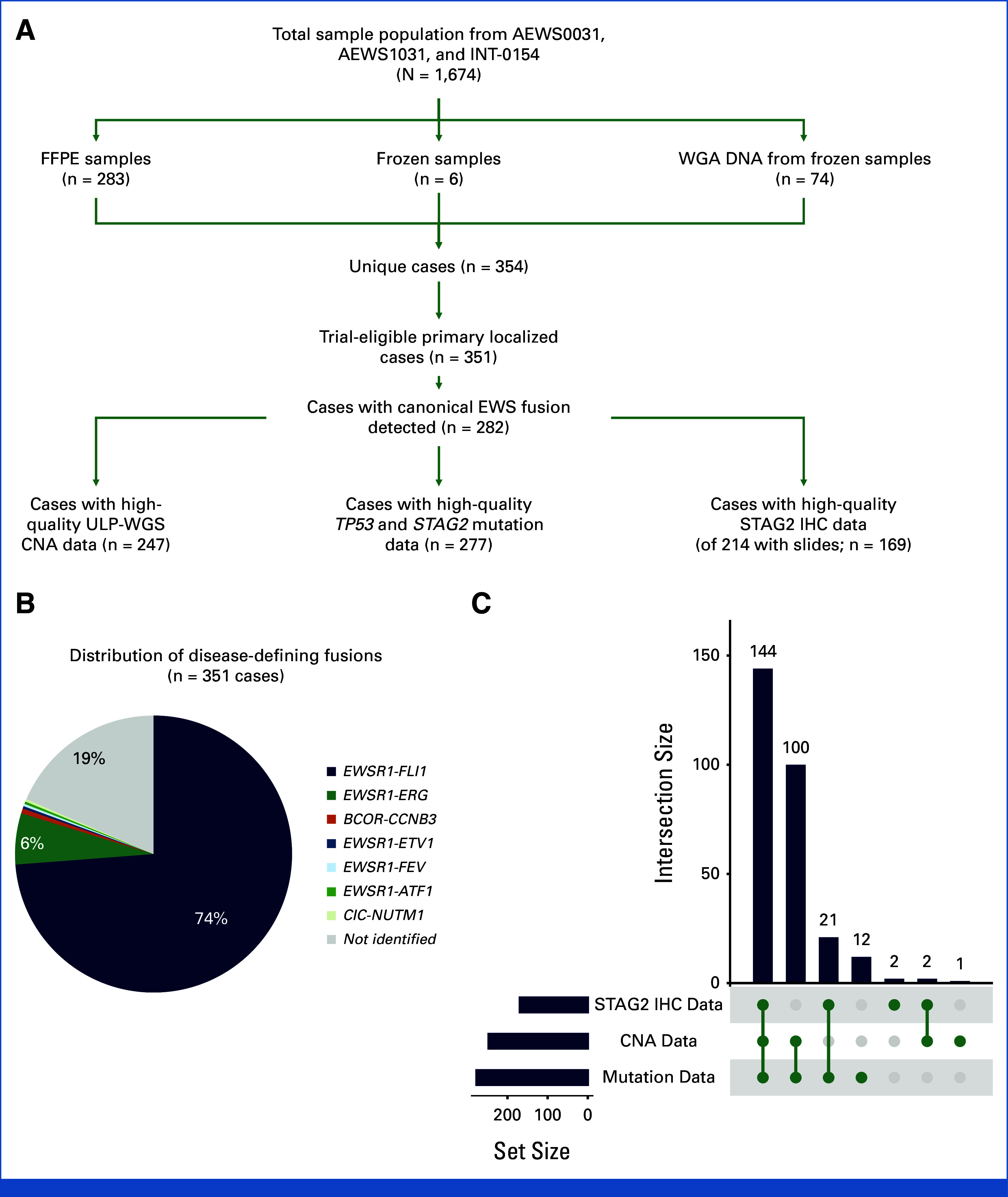
Cohort and study overview. (A) Flow diagram outlining source studies and patient counts. Among 351 eligible patients with viable tissue, 282 patients were found to have canonical EWS fusions and included for further study. (B) The most common canonical EWS fusion identified was *EWSR1-FLI1* followed by *EWSR1-ERG*. A total of 19% of patients had no fusion detected. (C) Large subsets of the analytic cohort were characterized by multiple molecular assays. CNA, copy number alteration; EWS, Ewing sarcoma; FFPE, formalin-fixed paraffin-embedded; IHC, immunohistochemistry; ULP-WGS, ultralow pass whole-genome sequencing; WGA, whole-genome-amplified.

Of 351 unique trial-eligible patients (Fig 1A), 282 patients (80.3%) were found to have a canonical EWS fusion of *EWSR1-FLI1*, *EWSR1-ERG*, *EWSR1-ETV*, or *EWSR1-FEV* (Fig 1B). No *FUS* fusions were detected. Of note, fusions associated with other round cell sarcomas were identified in four patients (1.1%). From the final analytic cohort of 282 patients with identified FET-ETS family fusions, 247 patients (87.6%) were evaluable for CNAs and 277 patients (98.2%) were evaluable for mutations. Two hundred fourteen patients had slides available for STAG2 IHC, and 169 patients (59.9%) had high-quality evaluable staining. Ninety-five percent of patients were represented with at least two molecular data modalities (Fig 1C).

In the final analytic cohort, 217 patients (77.0%) received treatment with interval-compressed chemotherapy, 251 (89.0%) patients were younger than 18 years at the time of trial enrollment, and there was a slight male sex predominance (53.9%, Table 1). There were no differences in clinical characteristics between the 282 patients with a canonical fusion in the analytic cohort and the 913 trial-eligible patients who were not included.

**TABLE 1.** Study Cohort Clinical Characteristics in Comparison With Unselected Patients

Variable	Not Included (n = 913), No. (%)	Included (n = 282), No. (%)	*P*
Sex			.588
Female	402 (44.0)	130 (46.1)	
Male	511 (56.0)	152 (53.9)	
Age, years			.059
<18	769 (84.2)	251 (89.0)	
≥18	144 (15.8)	31 (11.0)	
Race			.783
American Indian or Alaska	6 (0.7)	2 (0.7)	
Asian	21 (2.3)	5 (1.8)	
Black or African American	19 (2.1)	9 (3.2)	
Native Hawaiian or Other	10 (1.1)	2 (0.7)	
White	794 (87.0)	244 (86.5)	
NA	63 (6.9)	20 (7.1)	
Ethnicity			>.999
Hispanic or Latino	104 (11.4)	33 (11.7)	
Not Hispanic or Latino	793 (86.9)	246 (87.2)	
NA	16 (1.8)	3 (1.1)	
Tumor site			.639
Extraosseous	151 (16.5)	52 (18.4)	
Nonpelvic	605 (66.3)	179 (63.5)	
Pelvic	154 (16.9)	51 (18.1)	
NA	3 (0.3)	0 (0.0)	
Tumor volume, mL			.198
<200	393 (43.0)	147 (52.1)	
≥200	201 (22.0)	59 (20.9)	
NA	319 (34.9)	76 (27.0)	
Interval-compressed chemotherapy	694 (76.0)	217 (77.0)	.808
Study			<.001
AEWS0031	462 (50.6)	105 (37.2)	
AEWS1031	451 (49.4)	177 (62.8)	

Abbreviation: NA, not available.

### Univariate Analyses of Molecular Biomarkers

The 5-year cumulative incidence of relapse for the entire analytic cohort was 23.2% (95% CI, 18.4% to 28.4%), with a 5-year EFS of 73.5% (95% CI, 68.4% to 79.0%) and a 5-year overall survival (OS) of 81.4% (95% CI, 76.9% to 86.2%; Data Supplement, Appendix Fig A1).

**FIG 2.**
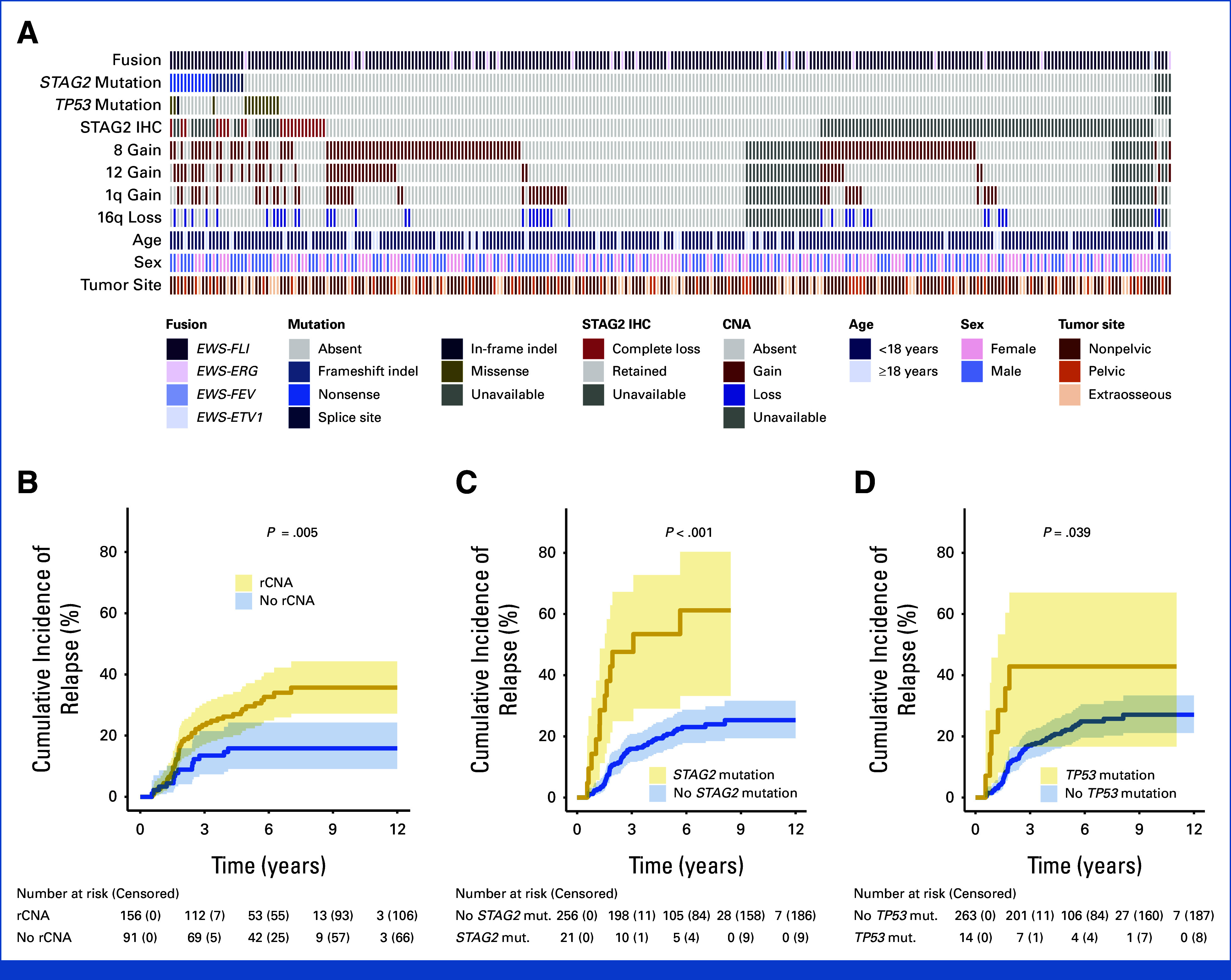
Molecular characterization of the cohort and cumulative incidence of relapse for rCNAs, *STAG2* mutation, and *TP53* mutation. (A) Comutation plot summarizing molecular data across the cohort of 282 primary localized Ewing sarcoma patients. Cumulative incidence of relapse for (B) rCNAs (defined as any of the four CNAs evaluated), (C) *STAG2* mutation, and (D) *TP53* mutation. CNA, copy number alteration; IHC, immunohistochemistry; mut., mutation; rCNA, recurrent copy number alteration.

Among 282 patients with a detectable fusion, *EWSR1-FLI1* was the most common fusion, seen in 259 patients (91.8%), followed by *EWSR1-ERG* seen in 21 patients (7.4%; Fig 2A). In a subset of 229 patients with *EWSR1-FLI1* fusions detected from DNA with unambiguous intronic breakpoints, Type I (57.2%) and Type II transcripts (24.0%) were most common (Data Supplement, Appendix Fig A2A).^17,18^ There was no difference in cumulative incidence of relapse in patients with *EWSR1-FLI1* fusions versus those with *EWSR1-ERG* fusions (23.6%, 95% CI [18.5% to 29.0%] *v* 20.6%, 95% CI [6.1% to 40.9%]; Gray's test *P* = .5; Data Supplement, Appendix Fig A2B). Similarly, Type I *EWSR1-FLI1* fusions were not prognostic relative to all other *EWSR1-FLI1* fusion subtypes (27.6%, 95% CI [20.0% to 35.6%] *v* 21.3%, 95% CI [13.6% to 30.1%]; *P* = .2; Data Supplement, Appendix Fig A2C).

Pathogenic *TP53* and *STAG2* mutations were identified in 5.1% (14 of 277) and 7.6% (21 of 277) of patients, respectively (Fig 2A). All the *STAG2* mutations were frameshift or nonsense mutations, and the nonsense mutation p.R216* was recurrent (Data Supplement, Appendix Fig A2D top). The *STAG2* variant allele frequency (VAF) range for males was 6.3%-93.7% (median, 51.3%), and the VAF range for females was 9.6%-40.7% (median, 31.9%), suggesting subclonal inactivation in many cases. All *TP53* mutations were in the DNA-binding domain and previously reported to be pathogenic; several were recurrent, with *TP53* p.R273C being the most frequent (Data Supplement, Appendix Fig A2D bottom). While some *TP53* variants were likely to be subclonal, the high median VAF suggested that most events were more consistent with clonal events (VAF range, 13.5%-86.3%; median, 54.5%; Data Supplement, Appendix Fig A2E). Of the 247 patients evaluable for CNAs, there were 20.6% with chromosome 1q gain, 50.6% with chromosome 8 gain, 21.1% with chromosome 12 gain, and 17.0% with chromosome 16q loss. One hundred sixty-nine patients were evaluable for STAG2 IHC, and complete STAG2 loss by IHC (normalized H-score of 0) was seen in 22 (13%) patients (Fig 2A).

We assessed previously reported full chromosomal and arm-level CNAs as prognostic biomarkers (Data Supplement, Appendix Fig A2F). No single CNA reached statistical significance in univariate analyses (Data Supplement, Appendix Figs A3A-A3D). Patients with any recurrent CNA considered in aggregate (defined as any chromosome 1q gain, 8 gain, 12 gain, or 16q loss) had an increased cumulative incidence of relapse (29.6%, 95% CI [22.4% to 37.1%] *v* 15.9%, 95% CI [9.1% to 24.3%]; *P* = .005; Fig 2B). Given the previously reported co-occurrence of 1q gain and 16q loss,^16,19^ we also evaluated the prognostic value of the combined biomarker of 1q gain and/or 16q loss and found it to be similarly associated with an increased cumulative incidence of relapse (36.8%, 95% CI [25.1% to 48.5%] *v* 19.8%, 95% CI [14.2% to 26.1%], *P* = .006). Interestingly, recurrent CNAs were not associated with inferior EFS or OS (Data Supplement, Appendix Figs A3E and A3F). We found this to be due to a disproportionate occurrence of the competing event of secondary malignancy (not an event of interest in the cumulative incidence of relapse outcome measure) among patients without recurrent CNAs (Data Supplement, Appendix Fig A3G), an unexpected observation requiring prospective validation.

The 5-year cumulative incidence of relapse in patients with a *STAG2* mutation was 53.4% (95% CI [29.1% to 72.7%]), which was significantly higher than the cumulative incidence of relapse in patients without a *STAG2* mutation (20.8%, 95% CI [15.9% to 26.1%]; *P* < .001; Fig 2C). Of 67 relapses in our cohort with available *STAG2* mutation status, 41 events were metastatic relapses (61.2%), 13 events were combined local/metastatic relapses (19.4%), and 13 events were local-only relapses (19.4%). Among patients with metastatic relapse, we observed that *STAG2* mutation was associated with an increased rate of metastatic relapse compared with patients without an *STAG2* mutation (39.2% *v* 11.9%, *P* < .001, Gray's test; Data Supplement, Appendix Fig A4A). Among patients with local or combined relapse, there were no differences in relapse rates by STAG2 status (Data Supplement, Appendix Figs A4B and A4C) although these events were rare overall. *TP53* mutations were also associated with a higher cumulative incidence of relapse (42.9%, 95% CI [16.6% to 67.0%] *v* 22.2%, 95% CI [17.2% to 27.5%]; *P* = .039; Fig 2D). EFS and OS analyses were also performed for *STAG2* mutation and *TP53* mutation, supporting the prognostic significance of both biomarkers using these outcome measures (Data Supplement, Appendix Figs A4D-A4G).

### Multivariable Model and Focused Analysis of Patients Receiving Interval-Compressed Chemotherapy

We used a multivariable model to ascertain which features were most predictive of cumulative incidence of relapse when controlling for other factors. We observed that *STAG2* mutation (hazard ratio [HR], 3.52; 95% CI, 1.75 to 7.10; *P* < .001) was the only molecular feature that remained prognostic in the multivariable analysis in addition to the clinical variables of age (HR, 1.11; 95% CI, 1.06 to 1.17; *P* < .001) and interval-compressed chemotherapy (HR, 0.37; 95% CI, 0.22 to 0.63; *P* < .001; Table 2). The same trends were observed when accounting for missing data using multiple imputation (Data Supplement, Appendix Table A3).

**TABLE 2.** Multivariable Analysis of Association of Molecular and Clinical Characteristics With Cumulative Incidence of Relapse Across 244 Patients With Complete Data

Molecular/Clinical Characteristic	HR	95% CI	*P*
rCNA	1.79	0.96 to 3.33	.066
*TP53* mutation	1.35	0.41 to 4.48	.600
*STAG2* mutation	3.52	1.75 to 7.10	<.001
Interval-compressed chemotherapy	0.37	0.22 to 0.63	<.001
Age	1.11	1.06 to 1.17	<.001
Male sex (relative to female sex)	0.62	0.36 to 1.04	.072
Site (relative to nonpelvic)			
Extraosseous	0.43	0.19 to 1.02	.054
Pelvic	0.95	0.53 to 1.71	.900

Abbreviations: HR, hazard ratio; rCNA, recurrent copy number alteration.

To evaluate the association of *STAG2* mutation with clinical characteristics, we regressed the presence of mutation against selected clinical variables using a logistic regression model. We observed that there was no significant association between *STAG2* mutation and the tumor site, patient age, or patient sex, as shown in the Data Supplement (Appendix Table A4). However, we did observe a positive association between *STAG2* mutation and tumor volume, whereby tumor volumes ≥200 mL were associated with an 8.58-fold increase in the odds of an *STAG2* mutation. As an exploratory analysis, we performed an alternative version of the multivariable analysis, including tumor volume data where it was available (n = 177 patients contributed to this multivariable model). As shown in the Data Supplement (Appendix Table A5), *STAG2* mutation remained independently prognostic.

**FIG 3.**
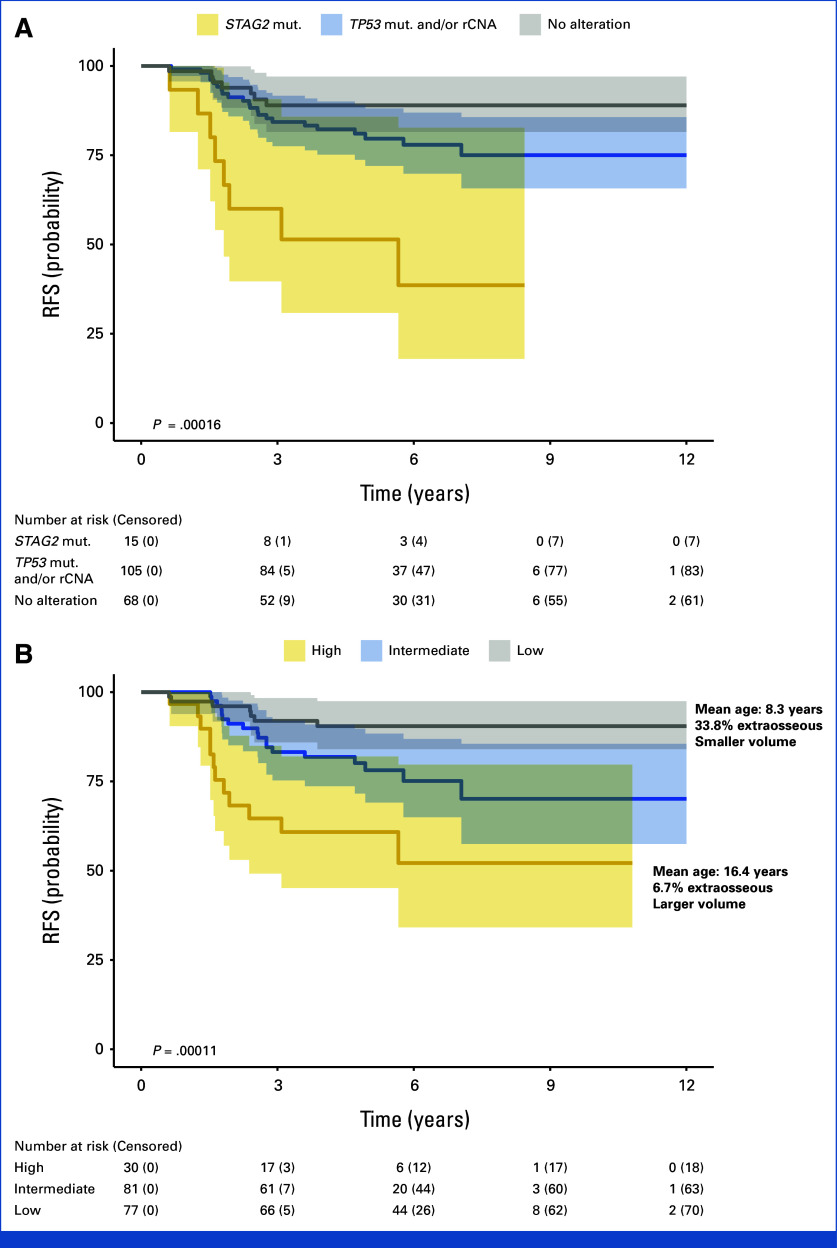
RFS when restricting only to patients who received interval-compressed chemotherapy. (A) RFS for three molecularly characterized groups in this cohort: (1) patients with *STAG2* mutation, (2) patients with *TP53* mutation and/or rCNAs but no *STAG2* mutation, and (3) patients with no identified molecular lesion. (B) When stratifying based on targeted RFS, low-risk patients were characterized by younger age, smaller tumor volumes, and a higher proportion of extraosseous tumors. mut., mutation; rCNA, recurrent copy number alteration; RFS, relapse-free survival.

To determine whether we could use the high-risk molecular and clinical features to identify prognostic risk groups, we used two approaches to risk stratify the patients included in the multivariable analysis who received interval-compressed chemotherapy (n = 188). We defined three molecular subgroups: (1) patients with *STAG2* mutation, (2) patients with *TP53* mutation and/or recurrent CNAs but no *STAG2* mutation, and (3) patients with no identified molecular lesion beyond the fusion and determined relapse-free survival (RFS) based on these groups. Using this approach, patients with *STAG2* mutation had increased risk of RFS event when compared with patients with no identified molecular lesion (5-year RFS 51.4%, 95% CI [30.8% to 85.8%] *v* 89.0%, 95% CI [81.5% to 97.0%]; Benjamini-Hochberg [BH]-corrected log-rank *P* < .001). Those with *TP53* mutation and/or recurrent CNAs without an *STAG2* mutation had a directionally increased risk of RFS event relative to those with no molecular lesion (79.6%; 95% CI, 72.0% to 88.1%), but this did not reach statistical significance (BH-corrected log-rank *P* = .096; Fig 3A). We also used an outcome-based approach in the interval-compressed chemotherapy cohort where low-, intermediate-, and high-risk groups were predefined according to RFS (Fig 3B). In addition to a lower prevalence of *STAG2* mutations as established by the multivariable analysis, low-risk patients were characterized by younger age (mean age, 8.3 years in low-risk patients *v* 16.4 years in high-risk patients), smaller tumor volumes (proportion of evaluable tumors with volume ≥200 mL: 15 of 69 [21.7%] in low-risk patients *v* 10 of 25 [40%] in high-risk patients), and a higher proportion of extraosseous tumors (26 of 77 [33.8%] *v* 2/30 [6.7%]). Taken together, *STAG2* mutation showed prognostic value after controlling for the receipt of interval compression and other clinical variables, and focused application of both risk group definitions to patients who received interval-compressed chemotherapy confirmed that *STAG2* mutation was the driving molecular feature for identifying patients with high risk of relapse, while also suggesting that clinical variables may be important for identifying the patients with the lowest risk of relapse.

### Composite Biomarker of STAG2 Loss by Mutation or IHC Is Associated With a Poor Outcome

Given the previous literature demonstrating that STAG2 loss of expression may occur with or without *STAG2* mutation,^11,13^ we evaluated STAG2 loss by IHC (Data Supplement, Appendix Fig A5A) and the composite biomarker of STAG2 loss by mutation or IHC in univariate analyses. Where there were overlapping molecular data, we observed a high concordance between *STAG2* mutation and STAG2 loss by IHC, with eight of 10 *STAG2* mutations among patients ascertained to have complete STAG2 loss by IHC (odds ratio for *STAG2* mutation 38.4, Fisher's exact *P* < .001; Data Supplement, Appendix Fig A5B). The *STAG2* mutation VAFs of the patients with complete STAG2 IHC expression loss versus those with partial STAG2 IHC expression loss were higher, but not significantly so (median, 47.9% *v* 25.4%; *P* = .27; Wilcoxon test). Restricting the analysis to males, this difference was greater, but still did not reach statistical significance (median, 80.5% *v* 25.4%; *P* = .1; Wilcoxon test). When restricting the analysis to females, there were insufficient patients for comparison. Interestingly, 14 patients with STAG2 loss by IHC had no *STAG2* mutations, supporting alternative mechanisms of *STAG2* inactivation in addition to mutation. Patients with STAG2 loss by IHC had a numerically but not statistically significantly higher cumulative incidence of relapse (32.6%, 95% CI [14.1% to 52.8%] *v* 17.4%, 95% CI [11.5% to 24.2%]; *P* = .075; Data Supplement, Appendix Fig A5C), and the composite biomarker of STAG2 loss by mutation or IHC was associated with a higher cumulative incidence of relapse (38.1%, 95% CI [21.9% to 54.2%] *v* 16.6%, 95% CI [11.0% to 23.5%]; *P* = .001; Data Supplement, Appendix Fig A5D).

To evaluate the additional prognostic value of STAG2 loss by IHC in isolation, we next evaluated the cumulative incidence of relapse associated with complete STAG2 IHC loss after removal of all pathogenic *STAG2* mutations. Of 155 patients with evaluable STAG2 IHC, we observed that there was no significant difference in cumulative incidence of relapse among patients with complete IHC loss (defined as a normalized H-score of 0) versus those with any retention of STAG2 IHC expression (14.9% *v* 16.6%, *P* = .9; Data Supplement, Appendix Fig A5E). Thus, while complete STAG2 IHC expression loss was highly associated with *STAG2* pathogenic mutation, exclusion of pathogenic *STAG2* mutations from outcome analysis suggests that complete STAG2 IHC expression loss does not show evidence of additional prognostic value in the current cohort.

Finally, we examined the relationship between hazard of relapse and normalized H-score as a continuous variable to provide some context around the threshold for use of *STAG2* IHC as a prognostic biomarker. Plotting the model-predicted relative hazard of relapse on the log scale by normalized H-score, we observed that a normalized H-score of 0 was predicted to have the highest hazard of relapse (Data Supplement, Appendix Fig A5F).

## DISCUSSION

We show in a large cohort of patients with newly diagnosed, molecularly defined, localized EWS treated with contemporary therapy while enrolled on prospective multi-institution therapeutic trials that molecular biomarkers identify populations of patients with high-risk disease. EWS is a disease defined by the presence of FET-ETS family fusions, and we were successfully able to detect such fusions in 80% of patients. A growing body of literature has suggested that inactivation of *STAG2* and *TP53* and multiple recurrent CNAs are associated with poor prognosis in EWS more generally.^12-16^ In the context of localized EWS, we confirm that the presence of recurrent CNAs and pathogenic *TP53* and *STAG2* mutations is individually associated with adverse outcomes and makes *STAG2* mutation a molecular biomarker for incorporation into future therapeutic trials. While we focused on cumulative incidence of relapse in this study, it is important to note that we observed a higher rate of secondary malignant neoplasms in patients whose tumors lacked molecular features associated with increased recurrence risk, at least partially explained by secondary malignancy and recurrence being competing risks.

A primary objective of the current study was to identify canonical fusions from newly diagnosed patients treated on prospective multi-institutional trials. While there has been mixed literature on the prognostic significance of various FET-ETS fusions within EWS,^20-23^ we found no significant difference in cumulative incidence of relapse for patients with *EWSR1-FLI1* versus *EWSR1-ERG* fusions, nor did we observe a difference in outcome for patients with Type I versus other *EWSR1-FLI1* fusions. We identified non–FET-ETS family fusions in a small subset of patients, which included patients with *BCOR-CCNB3*, *EWSR1-ATF1*, and *CIC-NUTM1*, all of which have differential outcomes.^24-26^ Among patients with no fusion identified, we hypothesize that the majority harbor FET-ETS fusions but had inadequate DNA or RNA for sequencing. This result underscores the importance of using a molecular approach to define EWS prospectively from high-quality tissue samples for prognostic biomarker research and, by extension, future clinical trials as well.

In 2014, three landscape analyses of EWS genomics identified correlations between alterations in *STAG2* and poor outcomes.^10-12^ Following this work, preclinical and clinical analyses have demonstrated the underlying biological and clinical impact of *STAG2* deleterious alterations in EWS, further supporting the notion that *STAG2* loss may contribute to the metastatic potential of EWS.^13,27,28^ In our current study of a large patient population enrolled on interventional trials, we found that *STAG2* mutation identified the highest-risk patients with a cumulative incidence of relapse of 53% (and an EFS of 47%), whereas patients without an *STAG2* mutation had a cumulative incidence of relapse of 21% (EFS of 76%). Importantly, *STAG2* mutation remained prognostic in a multivariable analysis, supporting the value of this molecular biomarker in identifying localized EWS patients with a higher cumulative incidence of relapse.

Multiple previous studies have suggested a prognostic impact of specific copy number gains and overall genomic complexity in EWS more generally.^12,15,16,29^ In our study, we evaluated specific recurrent CNAs in EWS: chromosome 1q gain, 8 gain, 12 gain, or 16q loss. When considered in aggregate, we observed that these recurrent CNAs were strongly associated with a higher cumulative incidence of relapse in localized EWS in univariate analyses. Previous studies have shown a consistent trend toward worse outcomes for patients with *TP53* mutations.^13,14^ In our current study, we observed that patients with *TP53* mutations in their diagnostic tissue have significantly inferior outcomes to those who are *TP53* wild-type in univariate analyses. However, the prognostic value of recurrent CNAs and *TP53* mutations was diminished in multivariable analyses, suggesting that both these molecular features co-occur with other clinical and molecular features of prognostic value in localized EWS.

Previous studies demonstrated that loss of STAG2 protein expression can occur without an identifiable *STAG2* mutation and is associated with poor outcomes in patients with localized EWS.^11,13^ We again identified patients with loss of STAG2 protein expression without an identifiable coding gene mutation in *STAG2*, providing further evidence that patients with EWS lose STAG2 expression through alternative means. Complete STAG2 loss by IHC trended toward an increased risk of relapse, and the composite biomarker of STAG2 loss by mutation or IHC was associated with an increased risk of relapse. However, we observed that complete STAG2 IHC expression loss excluding pathogenic *STAG2* mutations did not show evidence of additional prognostic value in the current cohort. It is possible that different aspects of STAG2 IHC staining, such as heterogeneity and patterns of staining that are not captured by the normalized H-score metric, might have additional prognostic value.^12^ However, we were limited in evaluating this further in this cohort given the technical challenges related to the use of archival tissue, interpretation of incomplete staining patterns, and the limitations of power. Taken together, these data support STAG2 loss by IHC as an emerging biomarker, warranting further technical refinement and future study.

There were several limitations to our study. The tissue obtained for sequencing included patients treated nearly 20 years before initiation of this study, and we were unable to detect fusions in approximately 20% of patients. This was likely due primarily to limitations in tissue quantity and quality but could have also been due to inclusion of fusion-negative small round blue cell tumors. Therefore, patients lacking detectable canonical EWS fusions were excluded from all biomarker evaluations. In addition, we were limited in the molecular biomarkers we were able to evaluate, with focal alterations in genes such as *CDKN2A* not reliably captured using ULP-WGS as an example. Despite these limitations, the molecular data for this study were generated from a median number of two unstained slides. These data pave the way for incorporation of molecular biomarkers into prospective clinical trials on which there would be significantly fewer tissue quality and quantity limitations.^30^

In summary, we found that patients with localized EWS and *STAG2* mutations were at the highest risk of relapse. Our findings support the incorporation of *STAG2* mutation and protein expression, *TP53* mutation, and CNAs into an integrated molecular biomarker strategy in future clinical trials to inform trial approaches testing treatment intensification and de-escalation. Ultimately, integrating clinical and molecular features will enable a further refinement of a risk stratification framework for localized EWS.

## Data Availability

A data sharing statement provided by the authors is available with this article at DOI https://doi.org/10.1200/JCO-25-00157.

## PREPRINT VERSION

Preprint version available on medRxiv (https://doi.org/10.1101/2025.01.20.25320840).
