# Supplementary material for: Molecular Characterization Informs Prognosis in Patients With Localized Ewing Sarcoma: A Report From the Children's Oncology Group
Source: J Clin Oncol. 2025 Nov 3;43(35):3750–9. doi: 10.1200/JCO-25-00157 (PMC12614438; doi:10.1200/JCO-25-00157)
Supplement: Supplementary file 2 [file jco-43-3750-s001.pdf]

ASCO Journals Data Sharing Statement Questionnaire

**1. Manuscript Title**	Molecular characterization informs prognosis in patients with localized Ewing sarcoma: A report from the Children's Oncology Group
**2. First Author Last Name**	Gillani
**3. Does your manuscript use ONLY data from a publicly available database (eg, SEER, Medicare)?** If "Yes," please specify the database; no additional information is needed. If "No," please complete questions 4-17.	No
**4. Will the data collected for your study be made available to others?** (If "No," enter context for your decision)	Partially. Concerns related to identification of patients limit the comprehensive release of all molecular and clinical data used in this study.
**5. List which data are available**	We are able to make deidentified/ aggregated molecular data used for this study available.
**6. Additional information about the data** (Enter “None,” if applicable)	None
**7. List how or where the data can be obtained** (e.g., e-mail address, URL, or other repository)	In the manuscript and associated supplementary materials.
**8. List beginning and end dates that data will be available** (e.g., MM/DD/YYYY to MM/DD/YYYY)	NA
**9. List any supporting documents** (Enter “None,” if applicable)	None
**10. Enter additional information about supporting documents** (Enter “None,” if applicable)	None

**11. How or where can supporting documents be obtained?** (e.g., e-mail address, URL, or other repository)	NA
**12. List beginning and end dates that supporting documents will be available** (e.g., MM/DD/YYYY to MM/DD/YYYY)	NA
**13. Indicate to whom data will be available** (Enter "U/K" for unknown; "N/A" for not applicable)	NA
**14. Indicate for what type of analysis or purpose** (Enter "U/K" for unknown)	UK
**15. Indicate by what mechanism** (Enter "U/K" for unknown)	UK
**16. Enter any other restrictions** (Enter "None," if applicable)	None
**17. Enter any additional information** (Enter "None," if applicable)	None

(Optional) If you have included or would like to include a Data Sharing Statement in addition to the above, please provide on the following page.

Data Sharing Statement
