# Supplementary material for: Molecular Characterization Informs Prognosis in Patients With Localized Ewing Sarcoma: A Report From the Children's Oncology Group
Source: J Clin Oncol. 2025 Nov 3;43(35):3750–9. doi: 10.1200/JCO-25-00157 (PMC12614438; doi:10.1200/JCO-25-00157)
Supplement: Supplementary file 3 [file jco-43-3750-s002.pdf]

Appendix A

Study Population

A total of 1,674 patients were enrolled on the parent trials AEWS0031, AEWS1031, and INT-0154. The initial analytic cohort included 354 unique patients from these trials with available FFPE (n=283), frozen tissue (n=6), or previously extracted WGA DNA (n=74). Two cases were subsequently excluded due to trial ineligibility and the single case enrolled on INT-0154 was removed.

Molecular confirmation of diagnosis was not required for trial eligibility. All patients were required to have newly diagnosed, localized EWS and available Formalin-Fixed Paraffin-Embedded (FFPE) tissue, frozen tissue, or previously sequenced whole-genome amplified (WGA) DNA from frozen tissue. Tissue was obtained from time of diagnosis in 95% of cases. The minimum quantity requested for FFPE material was two unstained slides. Among prospectively sourced patients enrolled to AEWS0031, preference was given to samples from patients who received interval compressed chemotherapy over non-interval compressed therapy. All patients signed informed consent at the time of enrollment to either AEWS0031, AEWS1031, or INT-0154. Separate approvals for this study were obtained from the Dana-Farber Cancer Institute Institutional Review Board, Children's Oncology Group (COG), and NCI.

Sample Preparation, DNA Extraction, and Sequencing

Fresh frozen tissue or FFPE was requested from the COG Biopathology Center (BPC), and DNA and RNA were extracted and quantified using standard methods. WGA DNA, generated from frozen tumor tissue for a previously published study, was available from a subset of patients treated on AEWS0031.[1] Ultra-low passage whole genome sequencing (ULP-WGS) and the TranSS-Seq assay were run on all samples as previously described.[2,3]

DNA was extracted at the BPC from frozen tumor tissue with the Qiagen Gentra Puregene Tissue kit or from FFPE tumor tissue (two unstained slides) using the Qiagen Allprep FFPE kit. DNA was quantified using PicoGreen, and libraries were prepared using KAPA HyperPrep Kit with Library Amplification (KAPA Biosystems, KK8504) and duplex UMI adapters (IDT). The mean DNA content extracted from cases was 519 ng (range 16-5,565 ng). Cases with any detected fusion had higher overall DNA content (median 389.4ng for fusion positive cases vs. 141.5ng for fusion negative cases; Wilcoxon $P < 0.001$) and mean target coverage (median 717.5x for fusion positive cases vs. 11.2x for fusion negative cases; Wilcoxon $P < 0.001$) suggesting limitations in comprehensive fusion detection from archival tissue with minimal DNA content where only two FFPE slides were available for most cases.

RNA was extracted at the BPC for a subset of cases using the Roche Highpure hybrid kit or by the Boston Children's Hospital Laboratory for Molecular Pediatric Pathology (LaMPP) using the Promega Maxwell RSC instrument and reagents or Covaris truXTRAC FFPE total NA Ultra Kit. Nucleic acid quantitation was performed with the Promega Quantus fluorometer using the RNA QuantiFluor kit.

Ultra-low passage whole genome sequencing (ULP-WGS) was performed on sequencing libraries to a target coverage of 0.1x (range <0.01x-0.74x, mean 0.18x). The ichorCNA algorithm was applied on the ULP-WGS BAM files and results were manually curated to determine copy number alterations.[2]

A custom hybrid-capture assay, TranSS-seq,[3] was run for all samples as previously described, with an anticipated mean target coverage of >150x (range <1x-3896x). The TranSS-seq assay has a validated bait set containing intronic regions of genes commonly involved in sarcoma

translocations, including *EWSR1* and *FUS*, as well as exonic regions of the genes *TP53* and *STAG2* (**Appendix Table A1**).

Identification of Gene Fusions

Gene fusions were identified using the published algorithms SvABA and BreaKmer.[4,5] Breakpoint coordinates were determined using UCSC Human BLAT Search with the hg19 assembly. Tumor content was quantified by comparing the translocation reads to wild-type reads with the following formula: % tumor DNA = T/[([W − T]/2) + T], where T is the number of translocation reads and W is the number of wild-type reads.

In the subset of cases with extracted RNA, fusion detection was performed using a custom fusion panel developed by LaMPP (**Appendix Table A1**). Total nucleic acid or RNA alone was isolated, RNA was converted to cDNA by reverse transcriptase, and library preparation was performed using a custom Archer FusionPlex kit (ArcherDX) using anchored multiplex PCR on an Illumina MiSeq sequencer. Sequencing reads were aligned, annotated and analyzed using the Archer Analysis bioinformatics software v6.2.7 system, as previously described.[6]

EWSR1-FLI1 fusion subtypes were categorized as Type I (defined as including the *EWSR1* transcript to exon 7 or 8 using the canonical transcript NM_005243/ ENST00000397938, and the *FLI1* transcript starting at exon 6 using the canonical transcript NM_002017/ ENST00000527786) or other to enable analysis of outcomes related to fusion subtype.[7] *EWSR1-FLI1* fusion subtype analysis was restricted to cases with DNA-based translocation calls, unambiguous intronic breakpoints, and only a single fusion subtype detected. Examples of DNA breakpoint data for *EWSR1-FLI1* and *EWSR1-ERG* were visualized using the St. Jude ProteinPaint web application (https://proteinpaint.stjude.org/).[8]

Mutation Identification and Annotation

TP53 and *STAG2* mutations were identified using the Mutect2 algorithm in "tumor-only" mode.[9] Mutations overlapping exons with allele fraction (AF) >= 0.05 and seen in at least three reads were retained as coding variants of possible significance. These candidate mutations were visualized using the Integrative Genomics Viewer (IGV version 2.3.81) and categorized as true positive or false positive calls depending on the depth of sequencing, the number of visualized alternative allele reads, the visualized AF, and the presence of artifacts at or around the examined mutation site. True positive mutations were subsequently curated for evidence of pathogenicity. *TP53* mutations with "Pathogenic" or "Likely Pathogenic" annotations in ClinVar were considered to be pathogenic in the somatic context and included in downstream analyses. *STAG2* mutations with nonsense, frameshift, or high impact splice site variants were considered to be pathogenic in the somatic context and included in downstream analyses. Mutation data for *TP53* and *STAG2* was visualized using the St. Jude ProteinPaint web application (https://proteinpaint.stjude.org/).

Because of limitations in estimating purity and ploidy with the sequencing modalities used in this study, we were unable to accurately estimate the cancer cell fraction (CCF) from the variant allele frequency (VAF) for *TP53* and *STAG2* mutations. Therefore, we focused on VAF analysis related to sub-clonality, acknowledging the limitation in its use as a proxy for the proportion of tumor cells in each sample with a mutation. Because *STAG2* is on the X chromosome, the distribution of VAF would be expected to be different in males vs. females, as has been described in the context of myeloid neoplasms previously.[10] Therefore, the distribution of VAF for *STAG2* was assessed in a sex-specific manner.

Identification of Copy Number Alterations

Based on prior evidence supporting the relevance of specific copy number alterations (CNAs) in Ewing sarcoma, chromosome 1q gain, chromosome 8 gain, chromosome 12 gain, and chromosome 16q loss were evaluated in this study. CNAs were identified using ".seg" files and manual review of tracings produced by the ichorCNA algorithm. Arm level gains and losses were called if greater than or equal to 50% of a chromosomal arm was altered. We assessed the relationship between each CNA and outcome separately and as a composite molecular feature of any of the four evaluated CNAs (collectively referred to as recurrent CNAs).

STAG2 Immunohistochemistry

Immunohistochemical staining was performed for STAG2 using the mouse anti-human monoclonal antibody (Santa Cruz SA-2 (J-12): sc-81852).[11]

Two pediatric pathologists evaluated intensity of nuclear staining across the total proportion of tumor cells visualized using the H-score, calculated as follows: (1 × percentage of weak staining) + (2 × percentage of moderate staining) + (3 × percentage of strong staining) within the tumor cells, ranging from 0 to 300. Due to variability in the staining of controls (endothelial cells), the H-score was subsequently normalized by comparing to maximum staining from the internal positive control, and capped at 300, as follows: Normalized H-score = min{300, (H-score)*3/(Highest staining from the internal positive control)}. Cases with inadequate viable tumor cells, extensive necrosis, features of harsh acid decalcification, or with no internal controls with observable STAG2 expression were considered uninterpretable.

Samples with normalized H-scores of 0 were determined to have "complete loss" of STAG2 expression, whereas samples with scores > 0 were considered to have at least partially retained expression. Samples determined to have complete loss of STAG2 expression were combined

with those identified as carrying loss-of-function *STAG2* mutations for the composite biomarker "STAG2 loss by mutation or IHC."

Statistical Methods

For descriptive analyses, categorical variables were presented as counts (percents) and continuous variables were summarized by medians. Associations between two categorical variables used the X^2 test or Fisher's exact test if any cell counts were less than five. The distributions of continuous, skewed molecular analysis quality control variables were compared using the Wilcoxon Rank Sum test.

Post-enrollment cumulative incidence of relapse was the primary outcome measure for this study. Second malignant neoplasms (SMNs) and deaths as first events were treated as competing risks. For univariate analyses, cumulative incidence curves were plotted and compared where appropriate using Gray's test for equality of cumulative incidence functions.[12] Supplemental analyses were also conducted for relapse-free survival (RFS), SMN-free survival (SMNFS), event-free survival (EFS), and overall survival (OS) using the Kaplan-Meier estimator and log-rank test for equality of survival curves. Patients who were not censored or who had not experienced a first relapse, SMN, or death event by twelve years after enrollment were censored at twelve years for visualization in Kaplan-Meier and cumulative incidence curves.

To estimate the association between molecular features and risk of relapse while controlling for clinical features, a multivariable Fine and Gray model for cumulative incidence of relapse was used.[13] Clinical investigators defined predictors to be included in the model prior to analysis of outcome data. These were determined by clinical importance and presence of missing data. If a potential prognostic factor had more than 25% missingness, it was removed from consideration

for inclusion in the final multivariable model. See **Appendix Table A2** for further details. This *a priori* variable selection approach was chosen due to the well-known limitations of stepwise variable selection and univariate screening.[14,15] As a sensitivity analysis, all primary univariate and multivariable cumulative incidence analyses were repeated using multiple imputation to account for missing data via the MICE algorithm.[16]

For the population of patients who received modern interval compressed chemotherapy and had molecularly defined EWS (i.e., the population that would be most similar to future trial populations), two different risk groupings were explored: (a) molecular and (b) multivariable model-derived. Three molecular subgroups defined (a): 1) patients with *STAG2* mutation, 2) patients with *TP53* mutation and/or recurrent CNAs but no *STAG2* mutation, and 3) patients with no identified molecular lesion. To visualize clinically meaningful risk groups from the multivariable model, which incorporates clinical risk factors as well as molecular, risk groups in (b) were based on target five-year RFS rates determined by clinical investigators as 90% for a low- and 50% for a high-risk group. Participants were ordered by their model-predicted risk of relapse and were sequentially added to low-risk and high-risk groups starting with ten patients (for estimation accuracy) until the target rate was exceeded. All remaining patients were considered to be intermediate-risk.

A two-sided *p*-value of ≤ 0.05 was considered significant in all analyses. Where pairwise comparisons were required between molecular risk groupings, the log-rank test p-values were adjusted for multiple comparisons using the method of Benjamini and Hochberg (BH) for controlling the false discovery rate.[17,18] Otherwise, no adjustment was made to account for the number of tests performed. All statistical analyses were performed using R Version 4.3.3 (R Core Team [2023]. R: A Language and Environment for Statistical Computing. R Foundation for Statistical Computing, Vienna, Austria. <https://www.R-project.org/>).

To evaluate the association of *STAG2* mutation with primary anatomic site, we used a multivariable logistic regression model to control for the impact of other potentially related clinical features (age at diagnosis, patient sex, and tumor volume). Of note, tumor volume was measured by retrospective chart review on AEWS0031 resulting in a large proportion of patients with missing tumor volume measurements from this cohort and measurement error concerns. Hence, tumor volume was analyzed as a categorical variable with traditional clinical cutoff ($\geq$ 200 mL vs. $<$200 mL).

To examine the relationship between hazard of relapse event and normalized H-score as a continuous variable, we fit a univariate cause-specific Cox proportional hazards models for hazard of relapse associated with normalized H-score. We modeled the relationship between hazard of relapse and normalized H-score using a restricted cubic spline with three basis knots equally spaced on the quantiles of normalized H-score to allow for nonlinearity in the relationship between the continuous variable and response.[19]

References

1 Lerman DM, Monument MJ, McIlvaine E, Liu X, Huang D, Monovich L, *et al.* Tumoral TP53 and/or CDKN2A alterations are not reliable prognostic biomarkers in patients with localized Ewing sarcoma: A report from the Children's Oncology Group. *Pediatr Blood Cancer* 2015;**62**:759–65. https://doi.org/10.1002/pbc.25340.

2 Adalsteinsson VA, Ha G, Freeman SS, Choudhury AD, Stover DG, Parsons HA, *et al.* Scalable whole-exome sequencing of cell-free DNA reveals high concordance with metastatic tumors. *Nat Commun* 2017;**8**:1324. https://doi.org/10.1038/s41467-017-00965-y.

3 Klega K, Imamovic-Tuco A, Ha G, Clapp AN, Meyer S, Ward A, *et al.* Detection of Somatic Structural Variants Enables Quantification and Characterization of Circulating Tumor DNA in Children With Solid Tumors. *JCO Precis Oncol* 2018;**2018**:1–13. https://doi.org/10.1200/po.17.00285.

4 Abo RP, Ducar M, Garcia EP, Thorner AR, Rojas-Rudilla V, Lin L, *et al.* BreaKmer: detection of structural variation in targeted massively parallel sequencing data using kmers. *Nucleic Acids Res* 2015;**43**:e19–e19. https://doi.org/10.1093/nar/gku1211.

5 Wala JA, Bandopadhayay P, Greenwald NF, O'Rourke R, Sharpe T, Stewart C, *et al.* SvABA: genome-wide detection of structural variants and indels by local assembly. *Genome Res* 2018;**28**:581–91. https://doi.org/10.1101/gr.221028.117.

6 Fisch AS, Church AJ. Special Considerations in the Molecular Diagnostics of Pediatric Neoplasms. *Clin Lab Med* 2022;**42**:349–65. https://doi.org/10.1016/j.cll.2022.05.007.

7 Gamberi G, Cocchi S, Benini S, Magagnoli G, Morandi L, Kreshak J, *et al.* Molecular Diagnosis in Ewing Family Tumors The Rizzoli Experience—222 Consecutive Cases in Four Years. *J Mol Diagn* 2011;**13**:313–24. https://doi.org/10.1016/j.jmoldx.2011.01.004.

8 Zhou X, Edmonson MN, Wilkinson MR, Patel A, Wu G, Liu Y, *et al.* Exploring genomic alteration in pediatric cancer using ProteinPaint. *Nat Genet* 2016;**48**:4–6. https://doi.org/10.1038/ng.3466.

9 Cibulskis K, Lawrence MS, Carter SL, Sivachenko A, Jaffe D, Sougnez C, *et al.* Sensitive detection of somatic point mutations in impure and heterogeneous cancer samples. *Nat Biotechnol* 2013;**31**:213–9. https://doi.org/10.1038/nbt.2514.

10 Katamesh B, Nanaa A, He R, Viswanatha D, Nguyen P, Greipp P, *et al.* Clinical and prognostic impact of STAG2 mutations in myeloid neoplasms: the Mayo Clinic experience. *Blood Adv* 2023;**7**:1351–5. https://doi.org/10.1182/bloodadvances.2022007937.

11 Shulman DS, Chen S, Hall D, Nag A, Thorner AR, Lessnick SL, *et al.* Adverse prognostic impact of the loss of STAG2 protein expression in patients with newly diagnosed localised Ewing sarcoma: A report from the Children's Oncology Group. *Br J Cancer* 2022;**127**:2220–6. https://doi.org/10.1038/s41416-022-01977-2.

12 Gray RJ. A Class of K-Sample Tests for Comparing the Cumulative Incidence of a Competing Risk. *Ann Stat* 1988;**16**:. https://doi.org/10.1214/aos/1176350951.

13 Fine JP, Gray RJ. A Proportional Hazards Model for the Subdistribution of a Competing Risk. *J Am Stat Assoc* 1999;**94**:496–509. https://doi.org/10.1080/01621459.1999.10474144.

14 Steyerberg EW. Clinical Prediction Models, A Practical Approach to Development, Validation, and Updating. *Stat Biol Heal* 2019. https://doi.org/10.1007/978-3-030-16399-0.

15 Jr. FEH. Regression Modeling Strategies, With Applications to Linear Models, Logistic and Ordinal Regression, and Survival Analysis. *Springer Ser Stat* 2015. https://doi.org/10.1007/978-3-319-19425-7.

16 Buuren S van, Groothuis-Oudshoorn K. mice : Multivariate Imputation by Chained Equations in R. *J Stat Softw* 2011;**45**:. https://doi.org/10.18637/jss.v045.i03.

17 Benjamini Y, Hochberg Y. Controlling the False Discovery Rate: A Practical and Powerful Approach to Multiple Testing. *J R Stat Soc: Ser B (Methodol)* 1995;**57**:289–300. https://doi.org/10.1111/j.2517-6161.1995.tb02031.x.

18 Yekutieli D, Benjamini Y. Resampling-based false discovery rate controlling multiple test procedures for correlated test statistics. *J Stat Plan Inference* 1999;**82**:171–96. https://doi.org/10.1016/s0378-3758(99)00041-5.

19 Harrell FE. *Regression modeling strategies*. vol. 54. New York: Springer-Verlag; 2001.

FIG A1

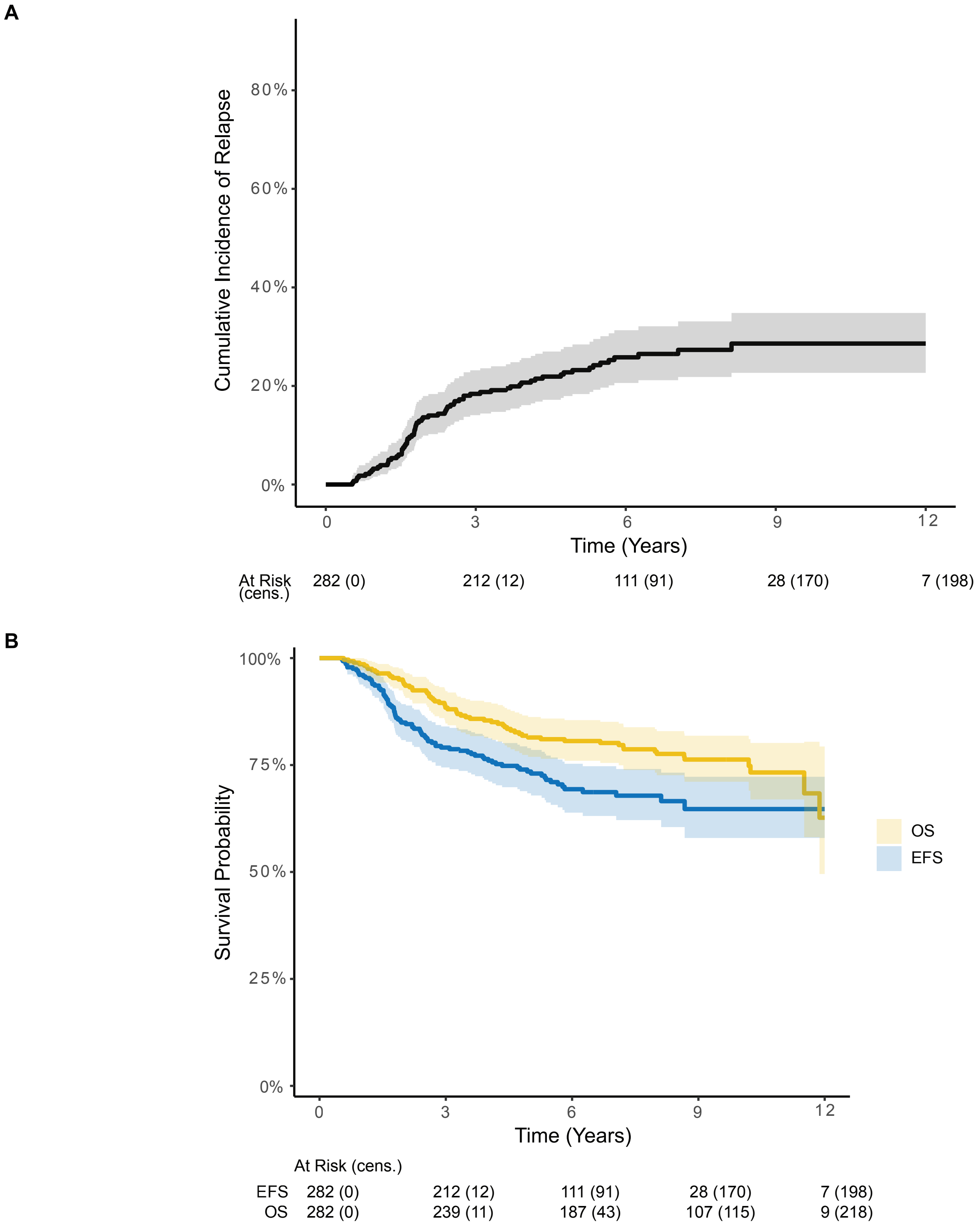

FIG A1. Overall outcomes in analytic cohort of 282 patients with localized EWS. (A) Cumulative incidence of relapse, (B) OS and EFS.

FIG A2

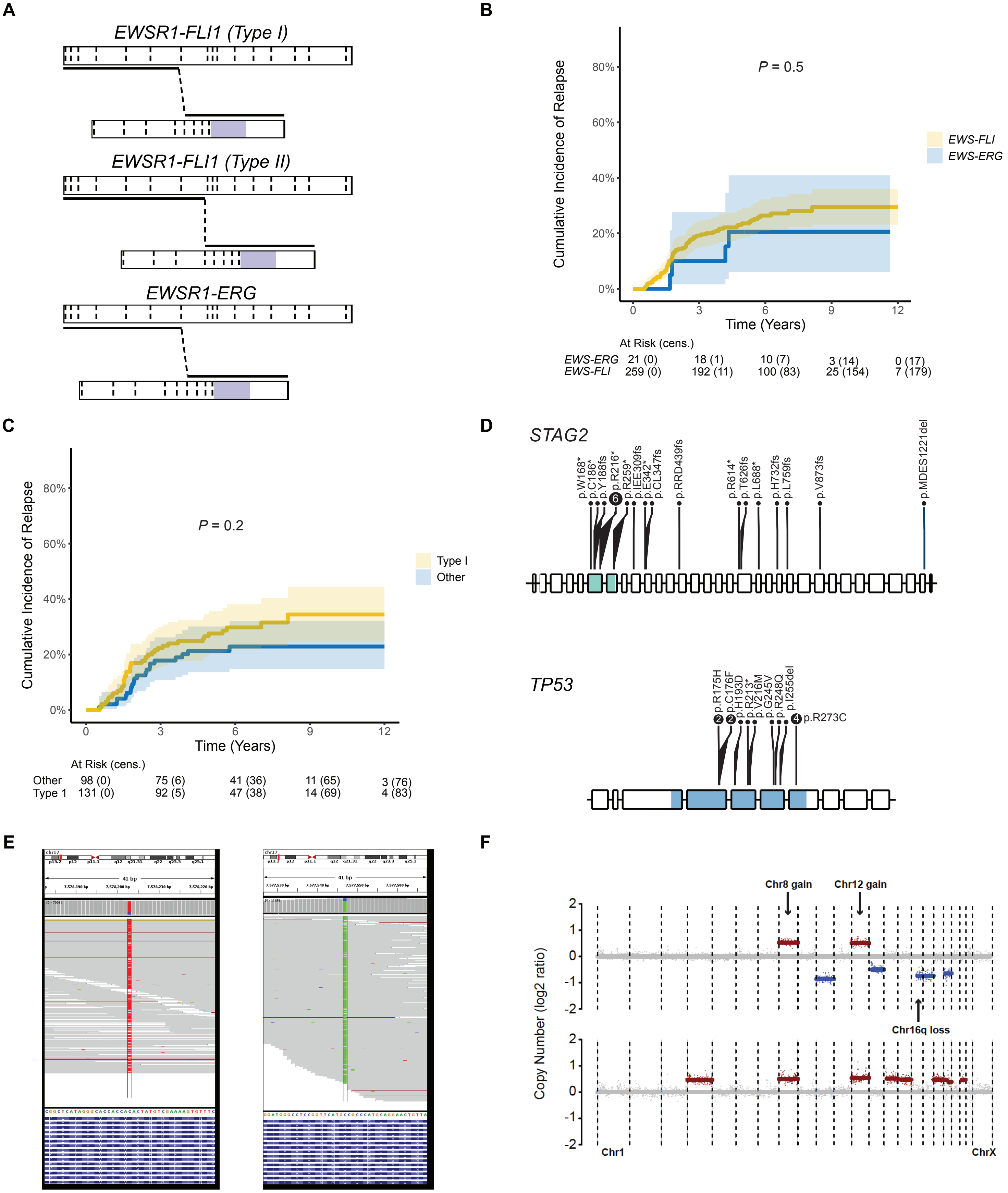

FIG A2. Selected visualizations and analyses related to fusions, mutations, and CNAs. (A) Illustrative examples of putative fusion transcripts of *EWSR1-FLI1* and *EWSR1-ERG* translocations observed in analytic cohort (purple = ETS domain). *EWSR1-FLI1* Type I fusions were defined as including the *EWSR1* transcript to exon 7 or 8 and the *FLI1* transcript starting at exon 6. *EWSR1-FLI1* Type II fusions were defined as including the *EWSR1* transcript to exon 7 or 8 and the *FLI1* transcript starting at exon 5. (B) Cumulative incidence of relapse for patients with *EWSR1-FLI1* vs. *EWSR1-ERG* fusions. (C) Cumulative incidence of relapse for patients with *EWSR1-FLI1* Type I vs. all other *EWSR1-FLI1* fusion subtypes. (D) Lollipop plots summarizing *STAG2* and *TP53* mutations in the analytic cohort (green = STAG domain, blue = P53 DNA-binding domain). Each lollipop circle represents one instance, unless otherwise specified by a number to indicate recurrence. (E) IGV screenshots representing high variant allele frequency mutations in *TP53*, suggesting loss of heterozygosity. (F) Representative ichorCNA tracings derived from ULP-WGS, used to evaluate chromosomal and arm-level CNAs in this study. Red = gain, blue = loss, gray = baseline copy number state, centered at log2 ratio = 0).

FIG A3

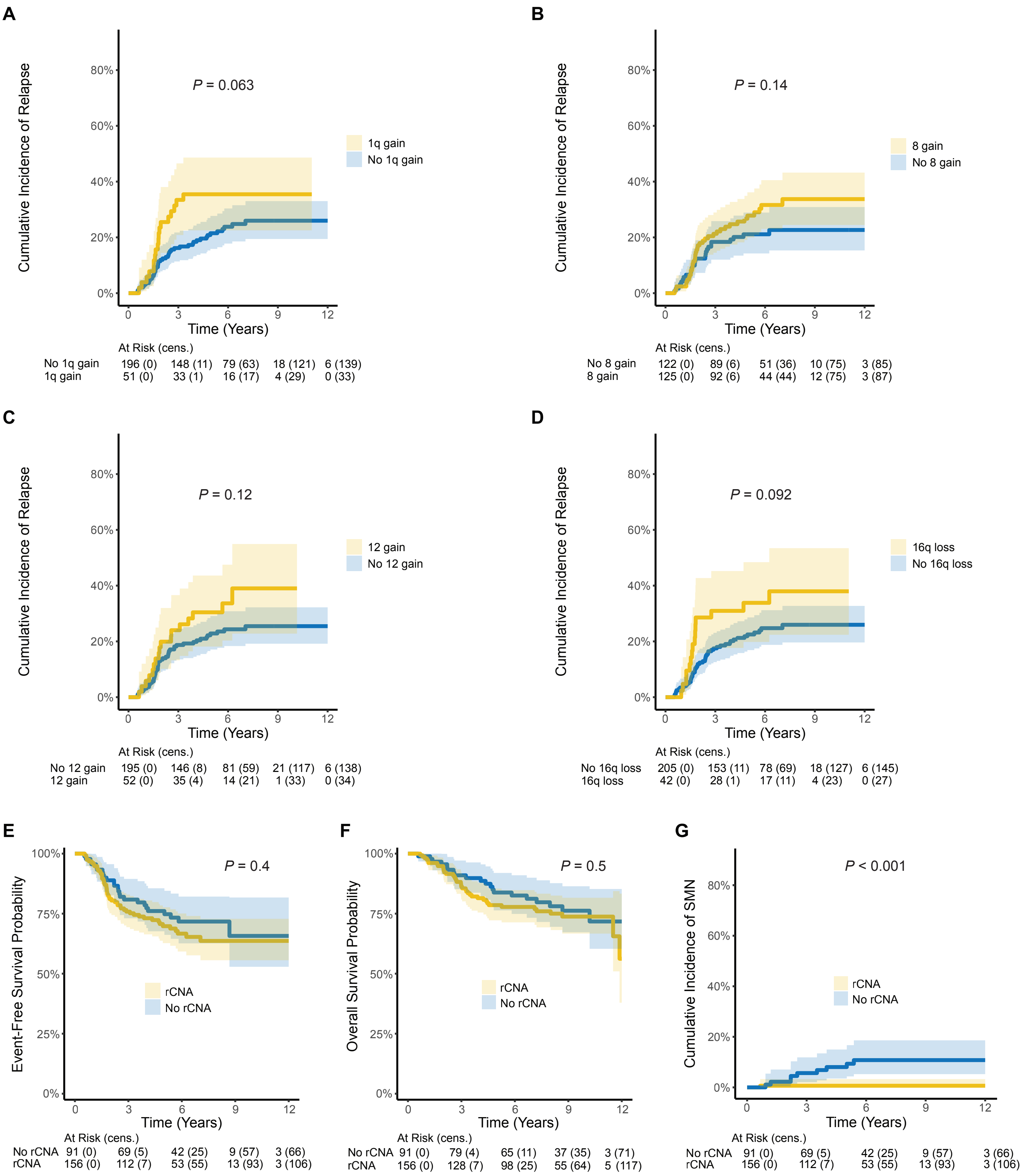

FIG A3. Outcomes related to recurrent CNAs. Cumulative incidence of relapse for (A) Chromosome 1q gain. (B) Chromosome 8 gain. (C) Chromosome 12 gain. (D) Chromosome 16q loss. (E) EFS for recurrent CNAs. (F) OS for recurrent CNAs. (G) There was an increased rate of secondary malignancies among patients without recurrent CNAs, explaining the apparent discrepancy between the outcome measures of cumulative incidence of relapse and EFS/OS for this biomarker.

FIG A4

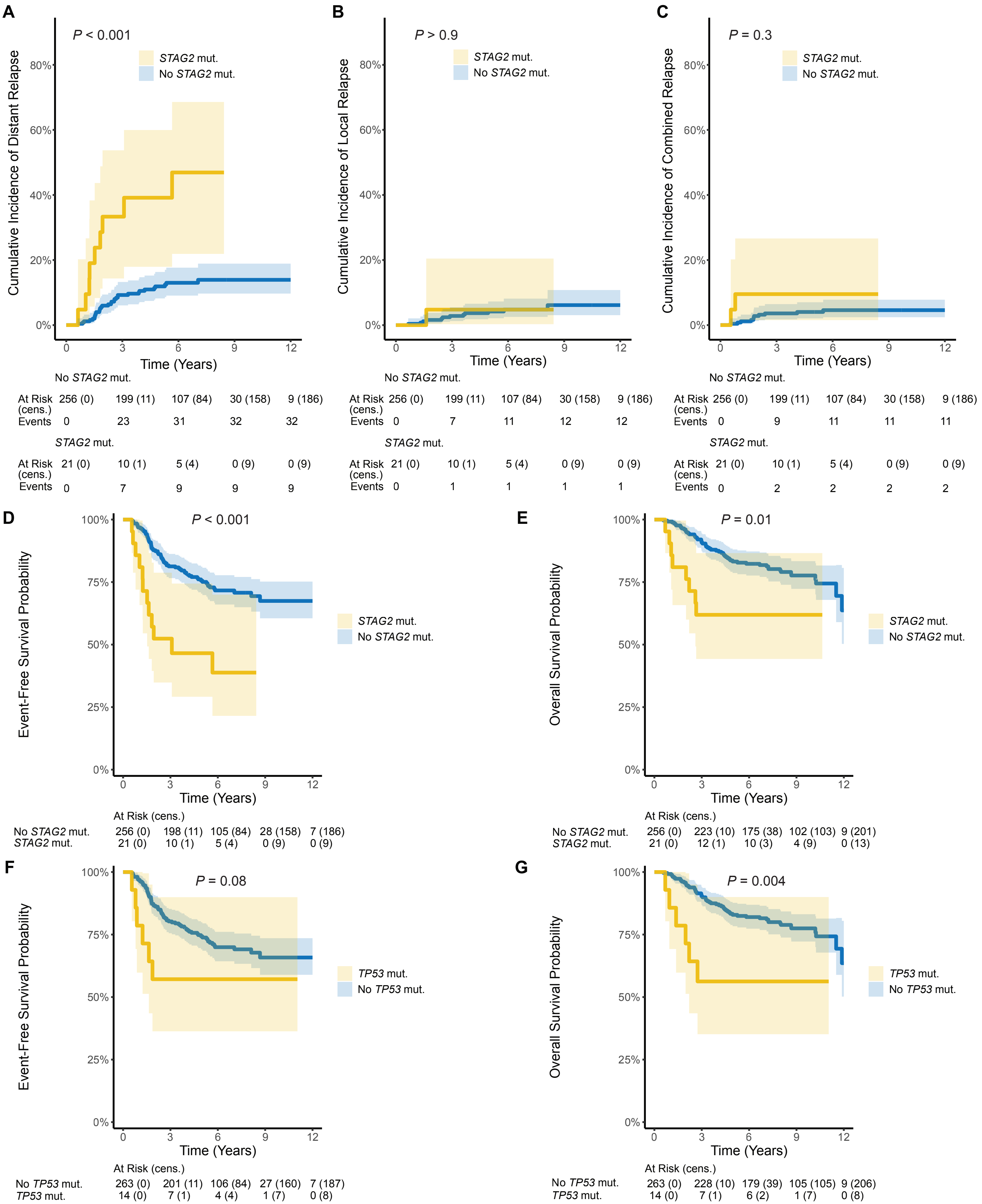

FIG A4. Outcomes related to *STAG2* mutation and *TP53* mutation. (A) Cumulative incidence of distant relapse for *STAG2* mutation. (B) Cumulative incidence of local relapse for *STAG2* mutation. (C) Cumulative incidence of combined local/distant relapse for *STAG2* mutation. *STAG2* mutation (D) EFS and (E) OS. *TP53* mutation (F) EFS and (G) OS.

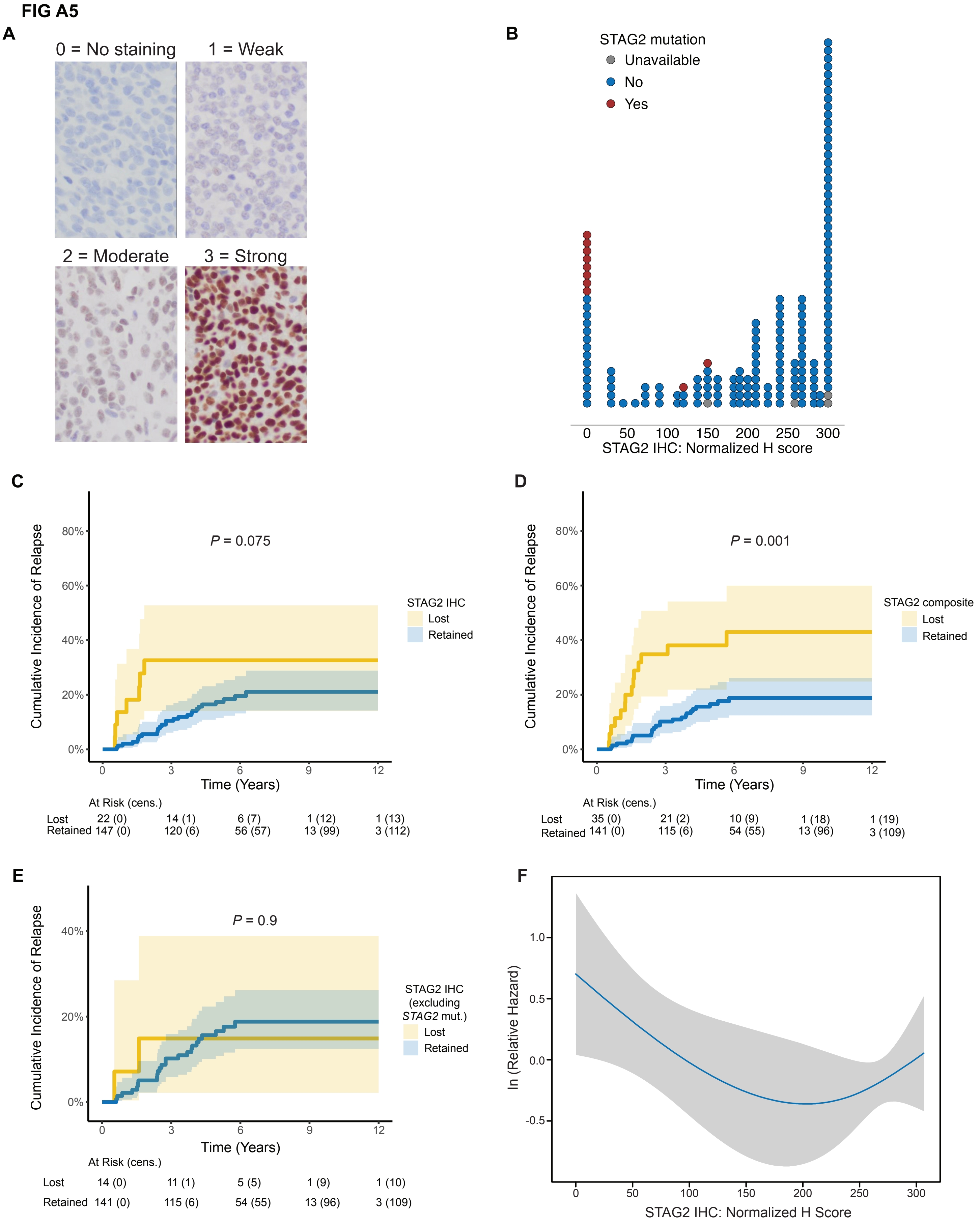

FIG A5. STAG2 immunohistochemistry (IHC). (A) Range of no nuclear staining to strong staining for STAG2 by IHC observed across the analytic cohort. The normalized H-score is based on the intensity of staining (as visualized here) as well as the proportion of cells with staining. (B) There was high concordance of *STAG2* mutation and complete loss of expression by IHC (defined by a normalized H-score of 0), and many samples had complete loss in the absence of a detectable *STAG2* mutation. (C) Cumulative incidence of relapse for patients with complete STAG2 loss by IHC vs. those without. (D) Cumulative incidence of relapse for patients with STAG2 loss by mutation or IHC vs. those without. (E) After excluding pathogenic *STAG2* mutations, there was no significant difference in cumulative incidence of relapse among cases with complete IHC loss vs. those with any retention of STAG2 IHC expression. (F) Evaluation of the range of normalized H-score values for STAG2 IHC supports a maximum predicted hazard of relapse at 0 (natural log scale).

TABLE A1

Genes included on custom panels

TranSS-seq	LaMPP				
BCOR	*ALK*	*ETV1*	*MEAF6*	*PPARG*	*TCF12*
CCNB3	*BCOR*	*ETV4*	*MET*	*PRKACA*	*TFE3*
CIC	*BRAF*	*ETV5*	*MKL2*	*PRKCA*	*TFEB*
DUX4L6	*BRD3*	*ETV6*	*MYB*	*PRKCB*	*TFG*
DUX4L7	*BRD4*	*EWSR1*	*MYBL1*	*PRKCD*	*USP6*
ERG	*C11orf95*	*FGFR1*	*NCOA1*	*QKI*	*VGLL2*
EWSR1	*CAMTA1*	*FGFR3*	*NCOA2*	*RAF1*	*YAP1*
FLI1	*CCNB3*	*FOSB*	*NTRK1*	*RELA*	*YWHAE*
FOXO1	*CIC*	*FOXO1*	*NTRK2*	*RET*	
FUS	*CDH11*	*FOXO4*	*NTRK3*	*ROS1*	
PAX3	*DNAJB1*	*FUS*	*NUTM1*	*SS18*	
PAX7	*EGFR*	*GLI1*	*PDGFB*	*SS18L1*	
STAG2	*EPC1*	*HMGA2*	*PHF1*	*STAT6*	
TP53	*ERG*	*MAML2*	*PLAG1*	*TAF15*	

TABLE A1. Genes included on the TranSS-seq assay and LaMPP fusion panel.

TABLE A2

Variable Considered	Degrees of Freedom Required	Include in Primary MV Model?	Notes
Age at diagnosis (years)	1	Yes	<25% missingness, prior evidence of association with risk of relapse
Ethnicity	1	No	Not a known prognostic factor associated with risk of relapse
Race	3+	No	Not a known prognostic factor associated with risk of relapse
Sex	1	Yes	<25% missingness, prior evidence of association with risk of relapse
Primary tumor site	2	Yes	<25% missingness, prior evidence of association with risk of relapse
Tumor volume (mL)	1	No	Prior evidence of association with risk of relapse but >25% missingness and non-uniform measurement on AEWS0031
Interval Compressed Chemotherapy	1	Yes	Experimental intervention on AEWS0031, applies to all patients on AEWS1031, proxy for modern therapy regimen
Therapy combination	1	No	VTC/VDC/IE vs. VDC/IE, showed no significant difference in risk of primary outcome measures per the AEWS1031 primary publication
Chemotherapy response (any vs. no viable tumor)	1	No	Only collected on the subset of patients who make it to the time point of local control event-free and receive surgery only or surgery followed by radiation therapy, not a known prognostic factor associated with risk of relapse
Type of local control	3	No	Only collected on the subset of patients who make it to the time point of local control event-free, not a known prognostic factor associated with risk of relapse
STAG2 loss by IHC	1	No	>25% missingness, investigated in a supplemental analysis
EWS Fusion	3	No	Not a known prognostic factor associated with risk of relapse, sparse categories outside of EWS-FLI1
TP53 Mutation	1	Yes	<25% missingness, primary molecular feature
STAG2 Mutation	1	Yes	<25% missingness, primary molecular feature
1q gain	1	No	Too many terms to support individually with limited dataset, insufficient prior literature demonstrating association with risk of relapse, include as a composite marker indicating whether any copy number change is present
8 gain	1	No	
12 gain	1	No	
16q loss	1	No	
rCNA	1	Yes	Composite indicator of whether any selected copy number alterations are present

TABLE A2. ***A priori*** **variable selection for multivariable model.**

TABLE A3

**Molecular/ clinical characteristic**	**HR**	**95% CI**	***P* value**
rCNA	1.82	0.97, 3.41	0.06
TP53 mutation	1.45	0.45, 4.71	0.534
STAG2 mutation	3.42	1.69, 6.92	<0.001
Interval compressed chemotherapy	0.4	0.24, 0.66	<0.001
Age	1.12	1.07, 1.17	<0.001
Male sex (relative to female sex)	0.61	0.37, 1.00	0.05
Site (relative to non-pelvic)			
Extraosseous	0.48	0.23, 1.01	0.052
Pelvic	1.01	0.57, 1.78	0.981

TABLE A3. Multiply imputed multivariable analysis of association of molecular and clinical characteristics with cumulative incidence of relapse.

TABLE A4

Association of *STAG2* mutations with clinical characteristics

Clinical characteristic	OR	*P* value
Age (years)	1.01	0.936
Male sex (relative to female sex)	0.78	0.696
Tumor volume ≥ 200 mL	8.58	0.002
Non-pelvic site (relative to extraosseous)	1.09	0.922
Pelvic site (relative to extraosseous)	1.08	0.939

TABLE A4. Results of a logistic regression model of *STAG2* mutation as a function of clinical variables.

TABLE A5

Molecular/ clinical characteristic	HR	95% CI	P value
rCNA	2.6	1.05, 6.41	0.038
TP53 mutation	0.94	0.22, 3.96	>0.9
STAG2 mutation	5.29	2.33, 12.0	<0.001
Interval compressed chemotherapy	0.22	0.11, 0.45	<0.001
Age	1.1	1.03, 1.17	0.003
Male sex (relative to female sex)	0.74	0.37, 1.50	0.4
Site (relative to non-pelvic)			
Extraosseous	0.45	0.18, 1.16	0.1
Pelvic	0.49	0.24, 1.03	0.059
Tumor volume ≥ 200 mL	1.27	0.61, 2.65	0.5

TABLE A5. Multivariable analysis of association of molecular and clinical characteristics with cumulative incidence of relapse, inclusive of tumor volume across 177 patients with complete data.
